# DNA from Slow-Growing Mycobacteria in Culture Negative Sputa Reveal Common Exposure to Mycobacteria

**DOI:** 10.3390/biology15070553

**Published:** 2026-03-30

**Authors:** Ramiro López-Medrano, Miriam Retuerto-Guerrero, Elizabeth de Freitas-González, Cristina Diez-Tascón, Carmen del Mar Pérez-López, Octavio Miguel Rivero-Lezcano

**Affiliations:** 1 Servicio de Microbiología Clínica, Complejo Asistencial Universitario de León, Gerencia Regional de Salud de Castilla y León (SACYL), Altos de Nava, s/n, 24071 León, Spain; rzlopez@saludcastillayleon.es; 2 Servicio de Reumatología, Complejo Asistencial Universitario de León, Gerencia Regional de Salud de Castilla y León (SACYL), Altos de Nava, s/n, 24071 León, Spain; mretuertog@saludcastillayleon.es; 3 Servicio de Neumología, Complejo Asistencial Universitario de León, Gerencia Regional de Salud de Castilla y León (SACYL), Altos de Nava, s/n, 24071 León, Spain; emfreitas@saludcastillayleon.es; 4 Servicio de Anatomía Patológica, Complejo Asistencial Universitario de León, Gerencia Regional de Salud de Castilla y León (SACYL), Altos de Nava, s/n, 24071 León, Spain; 5 Laboratorio de Hematología, Complejo Asistencial Universitario de León, Gerencia Regional de Salud de Castilla y León (SACYL), Altos de Nava, s/n, 24071 León, Spain; 6 Unidad de Investigación, Complejo Asistencial Universitario de León, Gerencia Regional de Salud de Castilla y León (SACYL), Altos de Nava, s/n, 24071 León, Spain; 7 Institute of Biomedical Research of Salamanca (IBSAL), 37007 Salamanca, Spain; 8 Institute of Biomedicine (IBIOMED), University of León, 24071 León, Spain

**Keywords:** colonization, nontuberculous mycobacterial pulmonary disease, *Mycobacteriales*, slow-growing mycobacteria, tuberculosis, sputum, PCR

## Abstract

Mycobacteria isolated only once in repeated sputum cultures are deemed colonizers that are not normally present in the lungs despite their ubiquitous presence in the environment. We have developed a sensitive and specific PCR assay to detect slow-growing mycobacteria in culture negative sputum and have found them in 30% of the samples obtained from individuals with clinical suspicion of tuberculosis or mycobacteriosis (*n* = 50) and in 45% of the samples from individuals with no suspicion (*n* = 49). As a negative control we have analyzed infections from an extra-pulmonary location, the urinary tract (*n* = 21), in which mycobacterioses are rare, and did not detect them. The load of bacteria in the lungs is kept low by the defense mechanisms of the host, and mycobacteria are not normally detected in sputum cultures. Their incidental isolation would not be a consequence of a new exposure to the microorganism but a sign of susceptibility to mycobacteriosis.

## Simple Summary

Mycobacteria comprise *Mycobacterium tuberculosis*, *Mycobacterium leprae* and several species of nontuberculous mycobacteria. The latter are environmental microorganisms that cause mycobacteriosis, a disease similar to tuberculosis. Mycobacterioses are chronic infections that require lengthy treatments, and their importance has increased in the last few decades. In this report, we show that they can be found in sputum from individuals with no clinical suspicion of tuberculosis or mycobacteriosis. Therefore, the development of a mycobacteriosis would not depend on a new exposure to the microorganism, because they may be normally present, but mainly on the susceptibility to the disease. This information is valuable for interpreting episodes of infection.

## 1. Introduction

Species that belong to the *Mycobacterium* genus are usually classified into three different groups; the *Mycobacterium tuberculosis* complex, which includes *M. tuberculosis*, *M. leprae* and *M. lepromatosis* (the leprosy bacteria), and the nontuberculous mycobacteria (NTM), which comprise the rest of mycobacterial species. From the microbiological point of view they are also subdivided into two groups: slow-growing mycobacteria (SGM) with species, such as *M. tuberculosis* or *M. avium*; and rapid-growing mycobacteria [1], such as *M. abscessus*. *M. tuberculosis* is an obligate pathogen that is transmitted from person to person, but NTM are ubiquitous environmental bacteria [2]. In the clinical microbiology laboratory, either the growth of a single colony of *M. tuberculosis* in solid media or the detection of growth in liquid media indicates that the patient has tuberculosis. In contrast, the interpretation of NTM growth is not so straightforward. The current guideline recommends for diagnosing NTM pulmonary disease that three criteria are met: clinical (pulmonary or systemic symptoms), radiologic (bronchiectasis with small nodules) and microbiologic (positive culture from at least two separate expectorated respiratory samples). In fact, some patients who only have a single NTM isolation have not shown evidence of progressive disease [3]. Therefore, these sporadic isolations are considered environmental contamination that may lead the clinician to conclude that the patient is not infected. To study the potential presence of mycobacteria in the lung, we have performed a molecular analysis to identify DNA in sputa from patients with no clinical suspicion of tuberculosis or mycobacteriosis and negative culture for mycobacteria.

## 2. Materials and Methods

### 2.1. Bacterial Strains

To determine the specificity of molecular methods, four bacterial groups, each represented by four clinical isolates from different species per group, were analyzed. The slow-growing mycobacteria (SGM) were *M. tuberculosis* (strain 48tb), *M. avium* (84av), *M. gordonae* (35gr) and *M. kansasii* (135kn). Rapid-growing mycobacteria were *M. abscessus abscessus* (12aba), *M. chelonae* (243ch), *M. mageritense* (110mg) and *M. fortuitum* (160fr). *Mycobacteriales* include several genera [4], and one species from each was selected: *Tsukamurella tyrosinosolvens* (73Tty), *Nocardia cyriacigeorgica* (70Ncy), *Gordonia bronchialis* (72Gbr) and *Corynebacterium striatum* (84Cst). Non-*Mycobacteriales* included *Pseudomonas aeruginosa* (5Pae), *Staphylococcus aureus* (16Sau), *Haemophilus influenzae* (85Hin) and *Streptomyces albidoflavus* (75Sal). Rapid-growing mycobacteria were grown for 3 days, and slow-growing mycobacteria for at least 7 days on Middlebrook 7H11 solid media at 37 °C. The remaining bacteria were grown overnight at 37 °C in either Tryptic Soy Agar or chocolate agar (*Haemophilus influenzae*).

### 2.2. Collection and Processing of Sputum Samples

A total of 120 samples were collected and divided into 3 groups:

**Group 1** comprised 50 sputum samples from patients under suspicion of tuberculosis. Most patients had clinical or radiological findings suggesting tuberculosis (7/50), hemoptysis (3/50), bronchiectasis (6/50) or chronic obstructive pulmonary disease (7/50). The bacterial groups most frequently isolated in sputum samples were enterobacteria (9/50), *Staphylococcus aureus* (4/50), and *Streptococcus pneumoniae* (3/50).

**Group 2** included 49 sputum samples from patients with no clinical suspicion of tuberculosis or mycobacteriosis, with other bacterial or viral infections and showing clinical or radiological findings suggestive of pneumonia or respiratory infection (20/49), exacerbation of chronic obstructive pulmonary disease (7/49) or bronchiectasis (4/49). In half of the samples, commensal microbiota were observed (17/49). In the remaining samples enterobacteria (6/49), *Staphylococcus aureus* (4/49), *Streptococcus pneumoniae* (3/49), or non-fermenting Gram-negative bacilli (*Pseudomonas*/*Stenotrophomonas*, 4/49) were isolated. When radiological studies were performed in patients with non-chronic pulmonary diseases, the patterns were characteristic of acute inflammation. No patients in this group had a history of tuberculosis or other mycobacterioses, and none were receiving immunosuppressive therapy.

**Group 3** included 21 urinary samples from patients suffering from urinary tract infections with more than 10^6^ bacterial colony forming units/mL. The most prevalent microorganisms were enterobacteria (20/21), mostly *E. coli* (11/21). In a single case, *Staphylococcus saprophyticus* was isolated.

Sputum samples were homogenized with dithiothreitol (Sputasol, Thermo Fisher, Waltham, MA, USA) and decontaminated with a final concentration of 1.5% NaOH for 15 min. Samples were neutralized with phosphate buffer, and one aliquot was inoculated in solid medium (Lowenstein Jensen) and another in automated liquid-culture medium, following the manufacturer’s instructions (Bactec MGIT, Becton Dickinson, Franklin Lakes, NJ, USA), for an incubation period of 6 weeks at 37 °C. The positivity and contamination proportions in liquid media were 2.2% and 6.3%, respectively. Samples with mycobacterial growth were excluded.

### 2.3. DNA Purification and PCR Amplification Assays

Samples (1080 μL) were precipitated with 10% 3 M sodium acetate (pH = 5.2, 120 μL) and 2.5 volumes of ethanol (3 mL) at −20 °C for 1 h, and centrifuged at 16,000× *g* for 20 min at 4 °C. The pellet was air-dried, and DNA was purified with a Maxwell 16 FFPE tissue LEV DNA purification kit (Promega, Madison, WI, USA). Eluted DNA (50 μL) was precipitated as before and the dried pellet resuspended in water (12 μL). We need also to consider whether NaOH decontamination may reduce the yield of recovered DNA, thereby influencing the number of positive samples detected. To evaluate this effect, we conducted an experiment comparing paired samples processed with and without NaOH. In one case, the signal was markedly stronger in the sample processed without NaOH, whereas the remaining samples showed no noticeable differences between treatments (Figure S1), suggesting that decontamination may have reduced detectability in some samples.

All PCR reactions were performed with the 2× MyTaq PCR mix (Bioline, London, UK). The quality of DNA for PCR amplification was assessed with *16S rRNA* primers designed for metagenomics studies in 10 μL reactions which contained 2 μL of DNA, as described elsewhere [5]. To rule out DNA degradation or the presence of PCR inhibitors, only samples that allowed the amplification of the corresponding 464 bp band were used for mycobacterial DNA analysis. Amplification with a panmycobacterial *16S rRNA* primer was performed in a 10 μL reaction with 3 μL of DNA, as described elsewhere [6]. To increase the specificity of the assay, four new primers in the same region amplified by the panmycobacterial *16S rRNA* [6] were designed using the Primer3web tool (version 4.1.0, https://primer3.ut.ee/). In a two-step PCR, two of the primers (outer primers) were used in a touchdown amplification protocol for *Mycobacteriales* DNA. The resulting amplicon was re-amplified using two additional primers in a nested PCR protocol specific for SGM *16S rRNA* (Table 1). The 5′ SGM *16S rRNA* primer was labeled with Atto633 to facilitate the visualization of the specific band. For all PCR reactions, a C1000 Thermal Cycler (BioRad, Hercules, CA, USA) was used. The touchdown protocol [7] was performed with a PCR mixture (6 μL) that contained 2× PCR mix (3 μL), outer primers (8 μM, 0.15 μL) and DNA (2.85 μL). Thermocycling parameters included an initial denaturation step at 95 °C for 1 min, followed by 10 cycles of 96 °C for 15 s, 68.9 °C (a subsequent drop of 0.5 °C per cycle) for 15 s, and 72 °C for 15 s. This was followed by 25 cycles of 96 °C for 15 s, 61.5 °C for 15 s, 72 °C for 15 s and a final extension at 72 °C for 10 min. The 96-well plate with the reactions was sealed with an adhesive PCR sealing foil sheet (ThermoFisher Scientific, Waltham, MA USA). When the reaction was finished, to prevent cross-contamination, the foil was perforated with a micropipette tip and the nested reaction mix was added for the second of the two-step PCR. The reaction mix was prepared with 2× PCR mix (5 μL) and SGM primers (0.64 μM, 5 μL) for a final volume in the well of 16 μL. The plate was again sealed with a new foil sheet, and thermocycling parameters included an initial denaturation step at 95 °C for 2 min, followed by 30 cycles of 96 °C for 15 s, 60.0 °C for 15 s, and 72 °C for 15 s. The final extension step was performed at 72 °C for 10 min. Extreme care was taken to avoid cross-contamination. DNA purification from sputa and PCR reactions were performed in different rooms. A set of micropipettes was dedicated exclusively to prepare the reaction mixes that were dispensed in a Mini-V/PCR hood (Telstar, Terrassa, Spain), using only dual filter tips (Eppendorf, Hamburg, Germany). In every set of reactions a positive control (*M. tuberculosis* genomic DNA) and a negative control (DNAse free water) were included. Reactions were analyzed in agarose gels that included the Perfect 100 bp DNA ladder (EURx, Gdańsk, Poland). Bands were stained with SimplySafe (EURx) or SybrGreen (Thermo Fisher) and visualized with a Molecular Imager FX Pro Plus (BioRad) using a 532 nm laser. The SGM *16S rRNA* band labeled with Atto633 was visualized with a 635 nm laser.

### 2.4. Cloning and Sequencing of the SGM 16S rRNA Amplicon

We obtained fresh DNA from five positive samples that had been frozen, and all of them provided the specific amplicon again. Sequences of restriction sites were added to the SGM *16S rRNA* primers. The *Eco*RI site was added to the forward primer (5′ TACAGAATTCCGAAGGTCCGGGTTCTCT 3′) and the *Xho*I site to the reverse primer (5′ ATATCTCGAGTCTCCCCTGCAGTACTCTAG 3′). After the PCR reactions, the specific bands were purified from the agarose gel, cloned into the multiple-cloning site of the pcDNA3 vector (Invitrogen, Waltham, MA, USA) and sequenced. Sequence analysis was performed with BLASTn (v2.17.0, https://blast.ncbi.nlm.nih.gov/Blast.cgi, accessed on 27 March 2026) and CLUSTAL (2024, https://www.ebi.ac.uk/jdispatcher/msa/clustalo, accessed on 27 March 2026).

### 2.5. Statistical Analysis

Fisher’s exact test was utilized to determine the association between SGM *16S rRNA* presence and analyzed groups. A *p* value < 0.05 was considered significant.

## 3. Results

### 3.1. Specificity and Sensitivity of the Mycobacterial Two-Step PCR Reaction

**Figure 1:**
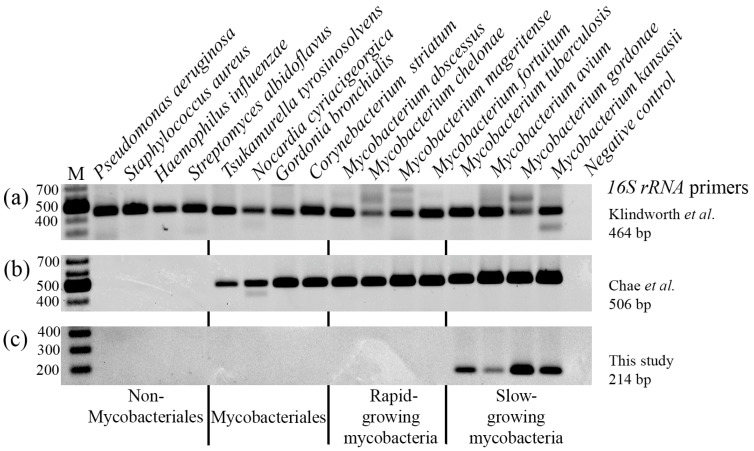
Specificity of SGM primers. DNA from clinical isolates of different bacterial species was amplified with increasingly specific primers [5,6]. (**a**) Amplification of DNA from all bacterial species. (**b**) Amplification from all *Mycobacteriales* species. (**c**) Amplification from slow-growing mycobacteria species. In the negative control, no bacterial DNA was added. M: molecular size ladder.

In a previous work, Chae et al. published a sequence of primers designed to detect panmycobacterial DNA by PCR [6]. We confirmed that these primers detected all the mycobacterial species that we analyzed (Figure 1b). Nevertheless, we also observed amplification of DNA from *Mycobacteriales* [4], a group of taxonomically related bacteria that included the *Mycobacterium* genus but also genera such as *Tsukamurella*, *Nocardia*, *Gordonia* or *Corynebacterium*. To increase the specificity of the assay, we designed two new sets of primers for performing nested PCR capable of amplifying DNA from SGM but not from rapid-growing mycobacteria, whose sequences were similar to those of other *Mycobacteriales* (Figure 1c).

**Figure 2:**
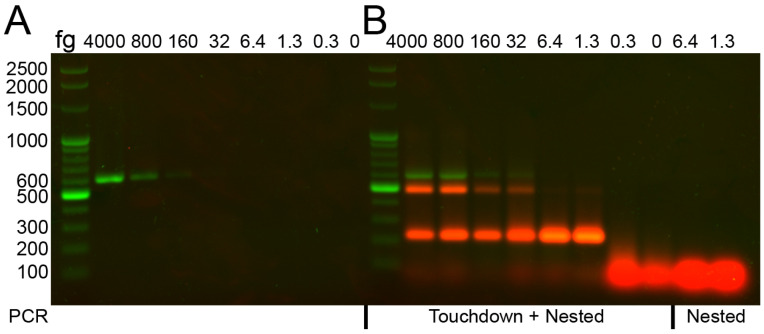
Sensitivity of SGM primers. (**A**) A dilution series was prepared with *M. avium* DNA from 4000 to 0.3 femtograms, which was amplified with set 5 primers (*IS1311*), specific to the *M. avium* complex. (**B**) The two-step PCR (touchdown + nested) amplified the same range of *M. avium* genomic DNA. In the last two lanes, the indicated amounts of DNA were amplified with only the second (nested) PCR.

Expecting a low number of mycobacterial DNA copies in culture-negative sputa, we tried to increase the sensitivity of the assay by using a touchdown protocol for the first reaction and a nested protocol for the second of the two-step PCR. To test the sensitivity of this protocol, we compared it with a published assay sometimes used to detect *M. avium* in the clinical setting [6], which employs primers that hybridize with *IS1311* (Table 1, set 5), a gene target specific to the *M. avium* complex, generating an amplicon of 600 bp. This band was not detectable below 160 fg (Figure 2A). Using the outer primers (Table 1, set 3) in the touchdown protocol, a band of 607 bp could be detected at 32 fg (Figure 2B, green band). Nevertheless, the subsequent nested PCR (Table 1, set 4) allowed the detection of the 214 bp band at 1.3 fg (Figure 2B, red band), showing that this protocol was at least 100 times more sensitive than the amplification of the *IS1311* target. When the outer primers could not generate an amplicon in the first PCR, the specific 214 bp band abruptly disappeared (Figure 2B, 0.3 fg). If the touchdown PCR step was not performed first, the primers from the nested PCR could not amplify the specific band directly from genomic DNA (Figure 2B, nested PCR), indicating the need to perform the two-step PCR (touchdown + nested PCR).

A nonspecific band of approximately 500 bp was also detected (Figure 2B, slower-migrating red band). The intensity of this band decreased when the genomic DNA was diluted. When this band was re-amplified, it yielded only the 214 bp product. Likewise, purification from agarose and subsequent sequencing again produced exclusively the 214 bp sequence. Together, these results indicated that the ≈500 bp band corresponded to the 214 bp amplicon migrating more slowly than expected, consistent with a conformational band shift. This anomalous mobility was likely caused by the high GC content of the fragment and the presence of several palindromic sequences capable of forming stable secondary structures. To test this hypothesis, the PCR product was heated at 95 °C in the presence of formamide. Under these denaturing conditions, the nonspecific band nearly disappeared, whereas the 214 bp band increased markedly in intensity, supporting the interpretation that the ≈500 bp band resulted from a conformational band-shift (Figure S2).

**Figure 3:**
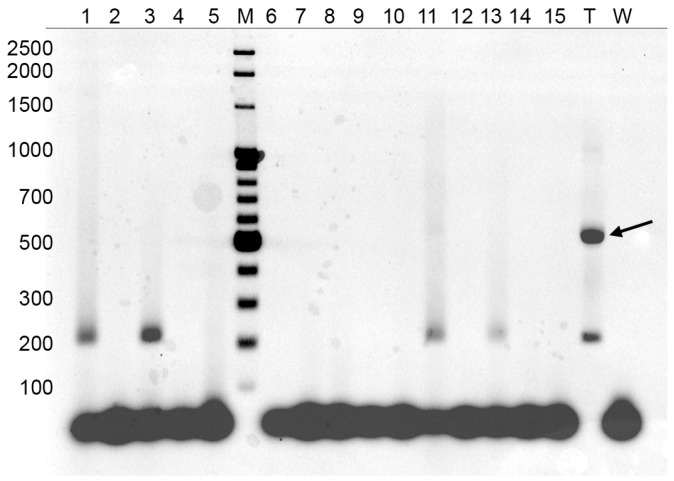
Two-step PCR performed with DNA purified from 15 samples of sputa. *M. tuberculosis* genomic DNA was used as the positive control (T) and water as the negative control (W). Arrow indicates the nonspecific band. M: molecular size ladder.

Figure 3 is an example of a two-step PCR reaction performed with DNA purified from sputa. The nonspecific band (arrow) was not apparent in the samples, but was prominent in the positive control (T). In this experiment, samples 1, 3, 11 and 13 contained detectable genomic DNA from slow-growing mycobacteria.

**Figure 4:**
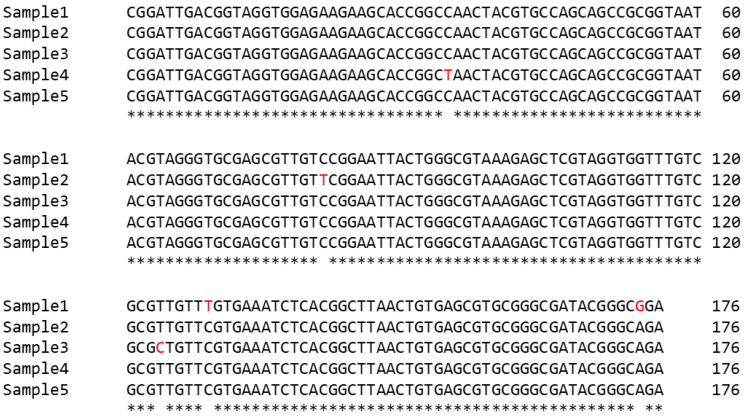
CLUSTAL sequence alignment of amplicons from five sputum samples. Nucleotide variations are highlighted in red. Primer sequences used in the nested PCR were omitted. Asterisks (*) indicate nucleotides that are completely conserved across the five sequences.

To confirm that the amplified 214 bp band corresponded to mycobacterial DNA, the amplicons from five sputum samples were cloned and sequenced. The sequence was highly conserved and matched the expected partial sequence of the *16S rRNA* gene of mycobacteria. Only five nucleotide positions showed variations (Figure 4, marked in red) among the different sequences. The hits with the highest scores in a BLAST analysis were mycobacterial species: *M. tuberculosis*, *M. szulgai*, *M. avium*, *M. kansasii*, *M. intracellulare*, *M. shottsii*, *M. paragordonae*, *M. bovis*, *M. marinum*, *M. gordonae*, *M. avium* subsp. *paratuberculosis*, *M. microti*, *M. arosiense* and *M. chimaera*. All species shared the same sequence in this region.

### 3.2. Detection of DNA from Slow-Growing Mycobacteria in Culture-Negative Sputa

Sputum samples were collected from three different groups of patients. Group 1 included patients with clinical suspicion of tuberculosis or mycobacteriosis. Group 2 included patients with clinical suspicion of pulmonary infections other than tuberculosis or mycobacteriosis. To verify that mycobacterial detection was largely confined to respiratory samples, we used urine samples from patients with urinary tract infections as negative controls, as this extrapulmonary site is seldom involved by mycobacteria and therefore should not produce a detectable signal with the PCR assay. In respiratory infections, DNA from SGM was detected in 30% of samples from group 1 and 45% from group 2 (Table 2). Although the number of positive samples was higher in group 2, there were no statistical differences between the two groups. This work proceeded in two stages. First, we analyzed samples from group 1 and handled samples stored at 4° C for two weeks. We then decided to study a second group and analyzed samples that had been at that temperature for only one week. This difference in storage time may have affected the detection rate of mycobacterial DNA, which could explain the lower number of positive samples in Group 1. As expected, no DNA from SGM was amplified in the negative control (group 3).

## 4. Discussion

Detection of *M. tuberculosis* by any bacteriological or molecular means determines treatment of the infection. Additionally, clinical signs compatible with tuberculosis also prompt the application of empirical treatments despite the risk of inappropriate treatment in patients without tuberculosis [8]. In contrast, detection of NTM does not strictly require the administration of antimicrobial agents. Current guidelines do not recommend treatment for patients in which NTM are detected only once in several sputum cultures [3], and the isolated mycobacteria is deemed to be a colonizer with no clinical relevance. Under the damage-response framework, colonization may result in an alteration of host homeostasis that does not result in enough damage to cause clinical disease [9]. The detection of mycobacterial DNA, unlikely in the very low doses present when a few bacilli are temporarily acquired from the environment, suggests that some multiplication has taken place and possibly colonized the host. Additionally, clinical disease may go undetected in asymptomatic patients. Pulmonary solitary nodules caused by NTM are incidentally found in asymptomatic patients and their surgical resection is usually curative [10]. Another clinical presentation that is closely associated with NTM is bronchiectasis [11], abnormal airway dilatations leading to chronic cough, sputum production and pulmonary infection. Some of these cases of bronchiectasis are also asymptomatic, for example in the elderly population or in rheumatologic patients [12,13]. Garcia et al. [14] investigated whether there were lung abnormalities in patients with colonizing mycobacteria, and they found features characteristic of bronchiectasis. As may be expected, radiological findings were worse for patients with NTM pulmonary disease than for colonized patients, with more lung lobes affected and greater frequency of cystic bronchiectasis [14]. Similarly, severity of tuberculosis assessed radiologically correlates with sputum bacillary load measured as colony-forming units/mL [15]. Fan et al. found that a substantial proportion of asymptomatic bronchiectasis (20%) end in clinical bronchiectasis after four years [16]. Although in this study no microbiological analysis was performed, this evolution may also take place in mycobacterial infections.

In the present article, we have gone one step further. We asked whether sputa of patients with no suspicion of tuberculosis or mycobacteriosis contained mycobacteria. With this objective, we designed an assay that is not intended for clinical use, since it cannot distinguish individual species; rather, it offers evidence of the degree of mycobacterial exposure, independent of the microorganism viability. Reports of diseases caused by uncommon mycobacterial species continue to appear regularly in the scientific literature. Notably, the genus *Mycobacterium* comprises more recognized pathogenic species than any other bacterial genus [17]. Our PCR assay was designed to detect any member of this diverse group regardless of its currently known clinical relevance. We restricted the analysis to samples that were negative for mycobacteria as determined by conventional microbiological methods. The presented results confirmed that many individuals are exposed to ubiquitous mycobacteria. The source of these mycobacteria in the sputa may be the lungs but also the oropharyngeal tract. Nevertheless, microaspiration may transport mycobacteria to the lungs, as has been suggested in patients with gastroesophageal reflux, a risk factor for NTM pulmonary disease [18]. Oral swabs have also been used to measure exposure to *M. tuberculosis* and have been proposed as a noninvasive alternative to sputum or bronchoalveolar lavage analysis [19]. Our results indicate that exposure to NTM is common and the development of the disease may depend more on host susceptibility than on new exposure in a naïve host. These microorganisms are characterized by exceptional durability and adaptability [20], and it seems reasonable to consider that if mycobacteria reach the lower airways, they may become part of the pulmonary microbiota without causing disease. They would not be isolated by conventional microbiological methods because the lung defense mechanisms will prevent their unrestricted multiplication and keep the mycobacterial population low and undetectable. In fact, most sputum samples processed in the microbiology laboratory ultimately yield negative culture results [21], and in our region, fewer than 4% of cultures are positive [22]. For this reason, we may need to reconsider the importance of what have been regularly deemed mycobacterial colonizers. It is possible that a single isolate indicates that mycobacterial growth is not being well controlled by the host defense mechanisms and that the patients have some degree of susceptibility to mycobacterial disease. A similar reflection has been made about the isolation of *Mycobacterium gordonae*. Generally regarded as a contaminant because it rarely causes infection, it has been observed that mycobacterial cultures from patients with bronchiectasis were more likely to grow *M. gordonae* than cultures from patients without bronchiectasis [23]. The authors of the study concluded that its isolation may suggest an increased risk of infection with more pathogenic NTM.

Exposure to environmental mycobacteria is common, as our results illustrate. Nevertheless, successful culture of these organisms from sputum samples is rare, most likely because their numbers in the lung are typically very low. When mycobacteria are isolated incidentally, it may reflect an increase in their abundance to a level detectable by culture, an event that could occur when the immune system fails to contain their replication. Such an inability to control mycobacterial growth may indicate an underlying susceptibility. In most individuals, these organisms persist harmlessly as colonizers; however, in others, underlying immune vulnerability may permit their progression to subclinical or asymptomatic infection and, in a minority of cases, to overt pulmonary disease, an outcome that may partly explain the current rising incidence of mycobacteriosis [24].

## 5. Conclusions

The presence of mycobacteria in sputum from individuals without suspicion of tuberculosis or mycobacteriosis suggests that exposure is frequent, but their low numbers prevent the detection by conventional microbiological protocols. Their isolation is unexpected in healthy individuals and their identification in the laboratory and classification as colonizers may have to be considered as a sign of susceptibility to mycobacteriosis, valuable information for the interpretation of potential future episodes of infection.

## Figures and Tables

**Table 1:** Primers used in the PCR reactions according to target bacterial *16S rRNA*.

Set	Bacterial Target	Primer Sequence (Forward and Reverse)	Reference
1	All bacterial species	5′ CCTACGGGNGGCWGCAG 3′	Klindworth [5]
		5′ GACTACHVGGGTATCTAATCC 3′	464 bp
2	All mycobacterial species	5′ GAGATACTCGAGTGGCGAAC 3′	Chae [6]
		5′ CAACGCGACAAACCACCTAC 3′	506 bp
3	*Mycobacteriales* (outer primers)	5′ ATGCAAGTCGAACGGAAAGG 3′	This study
		5′ CAGTCTCCCCTGCAGTACTC 3′	608 bp
4	Slow-growing mycobacteria (SGM)	5′ CGAAGGTCCGGGTTCTCT 3′	This study
		5′ TCTCCCCTGCAGTACTCTAG 3′	214 bp
5	*M. avium* complex (*IS1311*)	5′ TCGATCAGTGCTTGTTCGCG 3′	Chae [6]
		5′ CGATGGTGTCGAGTTGCTCT 3′	600 bp

**Table 2:** Detection of SGM *16S rRNA* in sputum.

Group	SGM *16S rRNA*	*p* Value ^1^
1 (*n* = 50)	15	
2 (*n* = 49)	22	0.149
3 (*n* = 21)	0	0.003 *

^1^ Groups 2 and 3 were compared with Group 1 by Fisher’s exact test. * *p* < 0.05.

## Data Availability

The original contributions presented in this study are included in the article. Further inquiries can be directed to the corresponding author.

## Supplementary Materials

The following supporting information can be downloaded at: https://www.mdpi.com/article/10.3390/biology15070553/s1, Figure S1: Effect of NaOH treatment on PCR yield; Figure S2: Denaturation of the PCR product with formamide.

## Abbreviations

The following abbreviations are used in this manuscript:

NTM	Nontuberculous mycobacteria
SGM	Slow-growing mycobacteria
PCR	Polymerase chain reaction
bp	Base pairs
DNA	Deoxyribonucleic acid
rRNA	Ribosomal ribonucleic acid
SDS	Sodium dodecyl sulfate
EDTA	Ethylene diamine tetraacetic acid
