# Supplementary material for: DNA from Slow-Growing Mycobacteria in Culture Negative Sputa Reveal Common Exposure to Mycobacteria
Source: Biology (Basel). 2026 Mar 30;15(7):553. doi: 10.3390/biology15070553 (PMC13071967; doi:10.3390/biology15070553)
Supplement: Supplementary file 1 [file biology-15-00553-s001.zip › biology-4190740-supplementary.pdf]

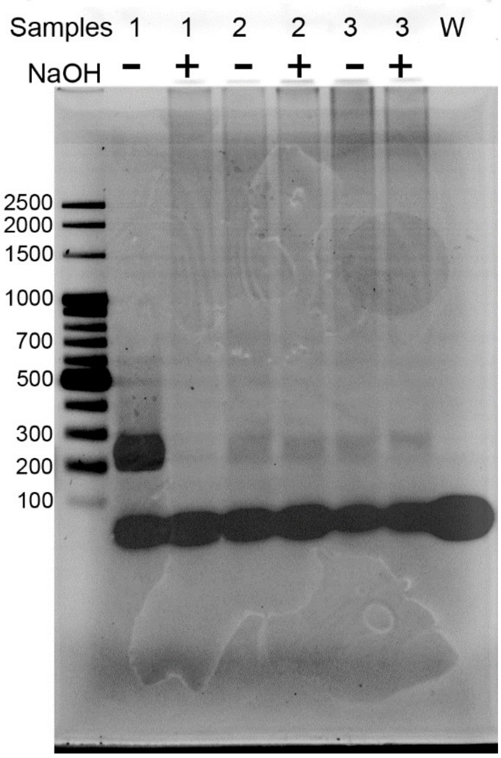

Figure S1. Effect of NaOH treatment on PCR yield. Three sputum samples were either decontaminated with 1.5% NaOH or processed without decontamination prior to DNA purification. The expected PCR product is 214 bp. Water was used as a negative control (W).

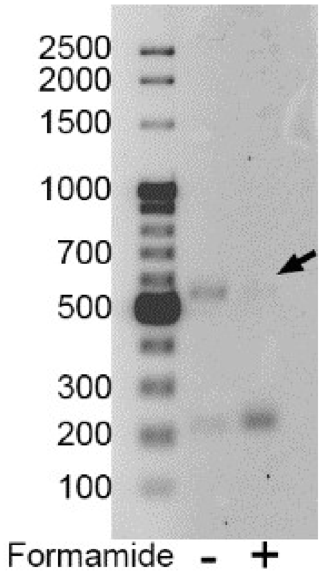

Figure S2. Denaturation of the PCR product with formamide. *M. avium* purified genomic DNA was used as the PCR template. The PCR product was mixed 1:1 with a denaturing formamide loading buffer (95% deionized formamide, 0.025% SDS, 10 mM EDTA), heated at 95 °C for 5 min, and subsequently chilled on ice for 2 min before electrophoresis. An untreated (non-denatured) PCR product was included as a control. The non-specific band is indicated with an arrow.
